# Differences in molecular characteristics and expression of virulence genes in carbapenem-resistant and sensitive *Klebsiella pneumoniae* isolates in Ningbo, China

**DOI:** 10.3389/fmicb.2024.1356229

**Published:** 2024-02-07

**Authors:** Min Jiang, Xuedan Qiu, Siyi Shui, Rongqing Zhao, Wenjun Lu, Chenyao Lin, Yanye Tu, Yifeng Wu, Qingcao Li, Qiaoping Wu

**Affiliations:** ^1^Department of Clinical Laboratory, The Affiliated LiHuiLi Hospital of Ningbo University, Ningbo, China; ^2^Department of Intensive Care Units, The Affiliated LiHuiLi Hospital of Ningbo University, Ningbo, China; ^3^Department of General Surgery, The Affiliated People’s Hospital of Ningbo University, Ningbo, China

**Keywords:** *Klebsiella pneumoniae*, carbapenem-resistant, hvKp, virulence, MLST

## Abstract

**Background:**

In recent years, *Klebsiella pneumoniae* has attracted attention because of its increasing drug resistance. At the same time, the migration and pathogenicity caused by its virulence genes also bring many difficulties to the diagnosis and treatment of clinical infections. However, it is currently unclear whether there are differences in virulence and pathogenicity with changes in drug resistance.

**Objective:**

To understand the differences in molecular characteristics and expression of virulence genes in carbapenem-resistant *Klebsiella pneumoniae* (CRKP) and carbapenem-sensitive *Klebsiella pneumoniae* (CSKP).

**Methods:**

Using polymerase chain reaction (PCR), we examined capsule polysaccharide-related genes and virulence genes in 150 clinical isolates of CRKP and 213 isolates of CSKP from the local area in Ningbo, China. Multilocus sequence typing (MLST) was used to analyze the phylogenetic relationships of clinical *Klebsiella pneumoniae* isolates. Furthermore, real-time quantitative PCR (RT-qPCR) was used to analyze the expression differences of common virulence genes in CSKP and CRKP, and the virulence was further verified by the larval model of *Galleria mellonella*.

**Results:**

The study found that the detection rates of genes *rmpA*, *iroB*, *peg-344*, *magA*, *aerobactin*, *alls*, *kfu*, and *entB* were significantly higher in CSKP compared to CRKP. The capsule gene types K1 and K2 were more common in CSKP, while K5 was more common in CRKP. Hypervirulent *Klebsiella pneumoniae* (hvKP) was predominantly from CSKP. CRKP strains exhibited noticeable homogeneity, with ST11 being the predominant sequence type among the strains. CSKP strains showed greater diversity in ST types, but ST23 was still the predominant sequence type. Carbapenem-sensitive hypervirulent *Klebsiella pneumoniae* (CS-hvKP) had higher expression of *rmpA* and *rmpA2* genes compared to carbapenem-resistant hypervirulent *Klebsiella pneumoniae* (CR-hvKP). In the wax moth virulence model, the survival rate of CS-hvKP was significantly lower than that of CR-hvKP.

**Conclusion:**

There is a significant difference in the distribution of virulence genes between CSKP and CRKP, with CSKP carrying a significantly greater number of virulence genes. Furthermore, compared to CSKP, CRKP strains exhibit noticeable homogeneity, with ST11 being the predominant sequence type among the strains. Additionally, in terms of virulence gene expression efficiency and virulence, CSKP is significantly higher than CRKP.

## 1 Introduction

*Klebsiella pneumoniae* (KP) is a Gram-negative rod-shaped bacterium belonging to the *Enterobacteriaceae* family. It is an opportunistic pathogen that is widely present in the human intestinal and respiratory tracts. KP can cause primary pneumonia or extrapulmonary infections when the host’s immune system is compromised, invasive procedures are performed, or antibiotics are used improperly, including bloodstream infections, urinary tract infections, meningitis, enteritis, liver abscesses, etc. (Choby et al., 2020; Wang et al., 2020; Zhang H. et al., 2021; Kuo et al., 2022). KP can be categorized into two types based on pathogenicity: classical *Klebsiella pneumoniae* (cKP) and hypervirulent *Klebsiella pneumoniae* (hvKP). Compared to cKP, infections caused by hvKP are more severe and widespread (Russo and Marr, 2019; Zhu et al., 2021a). HvKP exhibits stronger tissue invasiveness and cell migration, often resulting in concurrent infections in the bloodstream, eyes, liver, lungs, and central nervous system (Choby et al., 2020; Chang et al., 2021; Dai and Hu, 2022). The enhanced virulence of hvKP is mainly associated with factors such as capsular polysaccharides, mucoid phenotype regulatory genes, iron carriers, fimbriae, lipopolysaccharides, mobile genetic elements, etc. Most hvKP strains have a hypermucoviscosity phenotype (usually identified in string test), but it has been found that cKP can also exhibit hypermucoviscosity while string test of hvKP could be negative (Catalán-Nájera et al., 2017; Russo et al., 2018; Yang et al., 2020). In order to detect hvKP more accurately, in-depth studies have been conducted at the molecular level, using the five genes “*rmpA*, *rmpA2*, *peg-344*, *iucA*, *iroB*” for the diagnosis of hvKP with an accuracy of over 95% (Bulger et al., 2017; Russo and Marr, 2019). Capsular genes K1, K2, K5, K20, K54, and K57 have been shown to be highly associated with hvKP (Zhan et al., 2017; Russo et al., 2018; Yuan et al., 2019; Choby et al., 2020). With the horizontal transfer of virulence plasmids and carbapenemase-encoding plasmids (Gu et al., 2018; Zhang et al., 2020; Zhou et al., 2020), carbapenem-resistant hypervirulent *Klebsiella pneumoniae* (CR-hvKP) has been increasingly detected and even cause outbreaks in hospitals (Gu et al., 2018). In China, CR-hvKP has been detected in Zhejiang, Jiangsu, Beijing, Shanghai, Henan, Shandong, Hebei, and Inner Mongolia (Li et al., 2023; Pu et al., 2023; Yin et al., 2023). With the exponential increase in the detection of CR-hvKP, it poses a serious threat to public health. Due to the different selection of strains, the detection rate of virulence genes varied greatly in different selection studies. For example, the detection rate of *rmpA* gene can range from 2.2 to 87.2% (Lin et al., 2020; Li et al., 2023; Yin et al., 2023). Therefore, the differences in distribution and pathogenicity of virulence genes in CRKP and CSKP remain to be studied. Therefore, we used the five genes “*rmpA*, *rmpA2*, *peg-344*, *iucA*, *iroB*” as markers for hvKP and investigated the distribution and molecular epidemiological characteristics of virulence factors in CSKP and CRKP, as well as the differences in the expression levels of virulence genes and their pathogenicity in animal models.

## 2 Materials and methods

### 2.1 Bacterial strains and specimen source

In this study, a total of 363 strains of *Klebsiella pneumoniae* (KP) were selected from multiple hospitals located in Ningbo, Zhejiang Province, China, from January 2019 to December 2021. These included 150 strains of carbapenem-resistant *Klebsiella pneumoniae* (CRKP) and 213 strains of carbapenem-sensitive *Klebsiella pneumoniae* (CSKP). This study was approved by the Ethics Committee of Ningbo Medical Center LiHuiLi Hospital, Ningbo University (KY2023SL347-01). The specimens were obtained from various sources, including sputum, throat swabs, wound secretions, blood, and urine.

### 2.2 Identification of bacterial strains and drug susceptibility test

We used the VITEK 2 Compact automated system (bioMérieux, France) for bacterial strain identification and antimicrobial susceptibility testing. Data on drug susceptibility can be found in Supplementary Table 1. Carbapenem resistance was determined using the broth microdilution method. The quality control strains used for antimicrobial susceptibility testing were *Escherichia coli* ATCC 25922 and *Pseudomonas aeruginosa* ATCC 27853 (purchased from the National Center for Clinical Laboratories, Ministry of Health). The definition of carbapenem-resistant *Klebsiella pneumoniae* (CRKP) in this study was based on the MIC breakpoints specified by the Clinical and Laboratory Standards Institute (CLSI) in 2023, which indicate intermediate or resistant levels to one or more carbapenem drugs.

### 2.3 Detection of virulence genes and capsule typing genes

Genomic DNA was extracted from the samples using a boiling method (Liao et al., 2020b). Subsequently, polymerase chain reaction (PCR) was employed to amplify various genes related to capsule polysaccharide-related genes (*rmpA*, *rmpA2*, *magA*), iron carrier-associated virulence genes (*iucA*, *iutA*, *entB*, *aerobactin*, *iroB*, *ybts*, *kfu*), fimbriae-related genes (*fimH*, *markD*, *alls*), lipopolysaccharide-related genes (*wabG*), as well as inner membrane transport protein (*peg-344*) and capsular typing genes (K1, K2, K5, K20, K54, K57). The primer sequences and annealing temperatures for each gene were provided in Supplementary Table 2. The PCR reaction consisted of 12.5 μL of 2X Taq MasterMix, 1.0 μL of forward and reverse primers each, 2.0 μL of DNA template, and 8.5 μL of ddH_2_O, with a total volume of 25 μL. The PCR reaction conditions were as follows: initial denaturation at 94°C for 3 min, denaturation at 94°C for 30 s, annealing at the respective temperatures for 30 s, extension at 72°C for 1 min, with a total of 30 cycles, and a final extension at 72°C for 10 min. The PCR products were subjected to agarose gel electrophoresis on a 1.0% agarose gel at a voltage of 110 V for 30 min. Gel imaging and analysis were performed using the GELDOC XR gel imaging analysis system (Bio-Rad, USA), and the presence or absence of the target genes was determined based on the position of the marker bands. For positive bands, sequencing analysis was performed after gel purification.

### 2.4 Detection of hypermucoviscosity phenotype

The strains were inoculated onto Columbia blood agar plates using the streaking method in three zones. The petri dishes were then placed in a constant temperature incubator and cultured at 37°C with 5% CO_2_ for 18–24 h. A sterile disposable inoculation loop was used to pick up bacteria from a single colony and streaked upward. If the length of the mucoviscosity string observed during streaking was greater than 5 mm, and this phenomenon could be observed repeatedly more than 2 times, the string test result was considered positive, indicating that the strain was hypermucoviscosity *Klebsiella pneumoniae* (hmKP). Conversely, if the string test result was negative, the strain was considered non-hypermucoviscosity *Klebsiella pneumoniae* (n-hmKP).

### 2.5 Multilocus sequence typing (MLST)

Genomic DNA of *Klebsiella pneumoniae* was first extracted using a boiling method. PCR was then used to amplify 7 housekeeping gene fragments (*gapA*, *infB*, *mdh*, *pgi*, *phoE*, *rpoB*, *tonB*). The primer sequences and annealing temperatures for each gene can be found in Supplementary Table 1. The PCR reaction system consisted of 12.5 μL of 2X Taq MasterMix, 1.0 μL of forward and reverse primers each, 2.0 μL of DNA template, and 8.5 μL of ddH_2_O, with a total volume of 25 μL. The PCR reaction conditions were as follows: initial denaturation at 94°C for 5 min, denaturation at 94°C for 30 s, annealing at the respective temperatures for 45 s, extension at 72°C for 45 s, with a total of 35 cycles, and a final extension at 72°C for 5 min. The amplification products were sent to Shanghai Biotechnologies Corporation for sequencing. The sequencing results were uploaded to http://bigsdb.pasteur.fr/klebsiella/ and then compared with the database on the website to obtain the allele number and MLST type.

### 2.6 Relative expression levels of virulence genes

In this study, strains carrying the same virulence genes were selected from CSKP and CRKP, respectively, and their relative expression differences were analyzed using RT-qPCR. Details of the strains are shown in Supplementary Table 3. First, the Column Bacterial Total RNA Extraction Purification Kit (Bioteke, Shanghai) was used to extract RNA from the tested strains. Then, the One Step RT-qPCR Kit (Dye method) (Sangon Biotech, Shanghai) was used for RT-qPCR. The primer sequences can be found in Supplementary Table 1. Each gene was replicated three times. For data analysis, the 16S rRNA gene was used as the internal reference, and the 2^–△△CT^ method was used for calculation. Finally, GraphPad Prism 9.5 software (GraphPad Software, San Diego, CA, USA) was used for data visualization and analysis of relative gene expression levels.

### 2.7 Preparation of the infectious model using the *galleria mellonella* larvae

We used *Galleria mellonella* larvae as an infection model (Ménard et al., 2021; Asai et al., 2023). The strains were derived from RT-qPCR consistently, as detailed in Supplementary Table 3. *Galleria mellonella* larvae from Huiyude (Tianjin) weighing 250–350 milligrams, pale yellowish white in color, and exhibiting good activity and responsive behavior were selected as experimental subjects. Firstly, pilot experiments were conducted using control strains with pre-set concentrations of 10^7^ CFU/mL, 10^6^ CFU/mL, 10^5^ CFU/mL of injection solutions to determine the optimal concentration by observing larval mortality. The criteria for determining larval mortality were blackening of the body and no response to touch (Figure 7A shows survival and Figure 7B shows death). Ten larvae were injected for each strain, with the injection site being the rear lateral side of the second-last abdominal proleg, and care was taken to prevent any obvious leakage during the injection process. The injected larvae were placed in sterile disposable culture dishes and incubated in a lightproof incubator (Shanghai Yiheng) at 37°C. The survival of larvae was recorded every 6 h, and a survival curve was plotted. A saline control group was set up (10 larvae injected with 10 μL of physiological saline). The negative control strain was *Klebsiella quasipneumoniae* ATCC 700603, and the positive control strain was NTUH-K2044 (hvKP) (purchased from the National Center for Clinical Medicine Examination, Ministry of Health).

**FIGURE 1:**
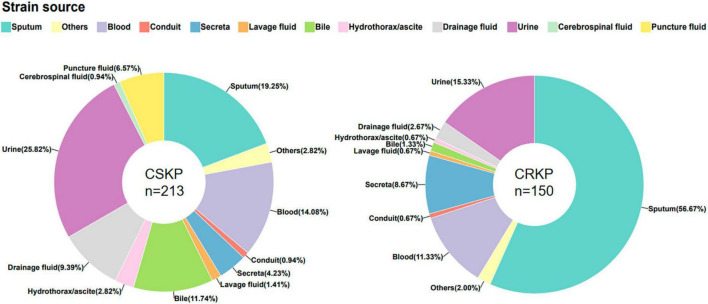
Source of CSKP and CRKP strains.

**FIGURE 2:**
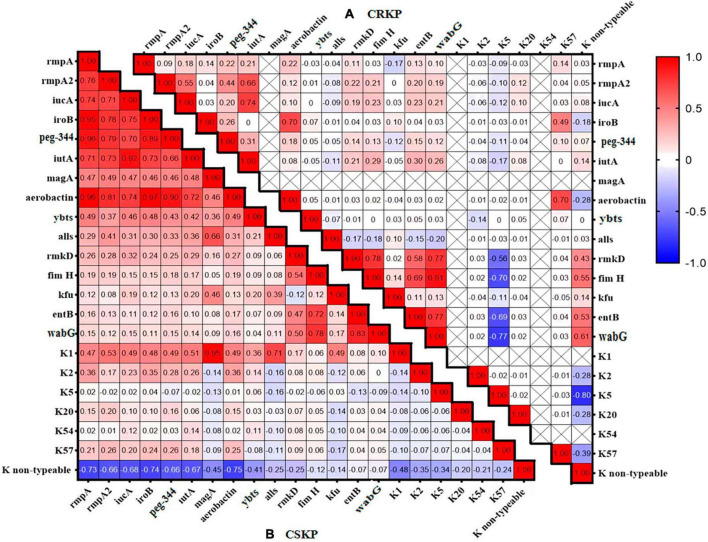
Heatmap of the distribution correlation of virulence genes. Panel **(A)** shows the correlation of virulence genes and capsule genes in CSKP, while Panel **(B)** shows the correlation in CRKP. The values in each square represent the correlation between the genes on the horizontal and vertical axes. Values closer to 1 indicate a stronger positive correlation, values closer to –1 indicate a stronger negative correlation, and values closer to 0 indicate no correlation. “ × ” indicates genes that were not detected and for which correlation cannot be calculated.

**FIGURE 3:**
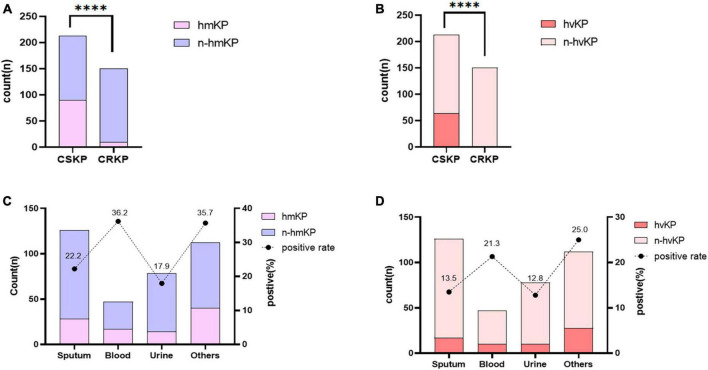
Differential distribution of high mucoid phenotype and high-virulence genes in different groups. Panel **(A)** shows the comparison of hmKP detection rates between CSKP and CRKP, while Panel **(B)** shows the comparison of hvKP detection rates. ****P < 0.0001. Panel **(C)** shows the comparison of hmKP detection rates between different sources, while Panel **(D)** shows the comparison of hvKP detection rates. The number indicates the positive rate of each group.

**FIGURE 4:**
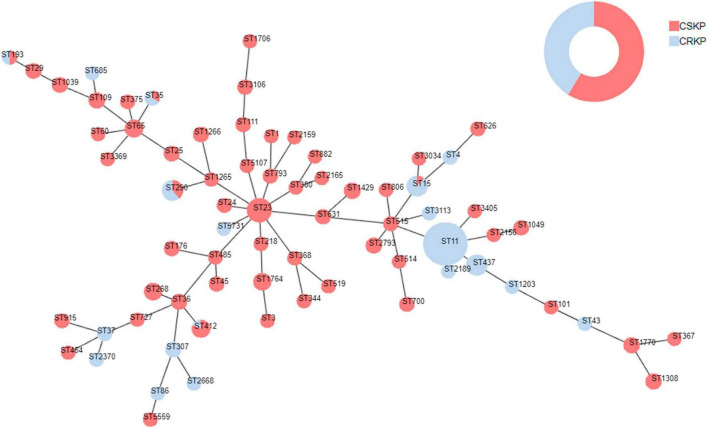
Minimum spanning tree of *Klebsiella pneumoniae*. Red represents CSKP, and blue represents CRKP. The minimum spanning tree is constructed using seven allelic genes (*gapA*, *infB*, *mdh*, *pgi*, *phoE*, *rpoB*, *tonB*) of *Klebsiella pneumoniae*. The size of the nodes is proportional to the number of isolates, and the length of the lines between nodes is proportional to the number of different alleles.

**FIGURE 5:**
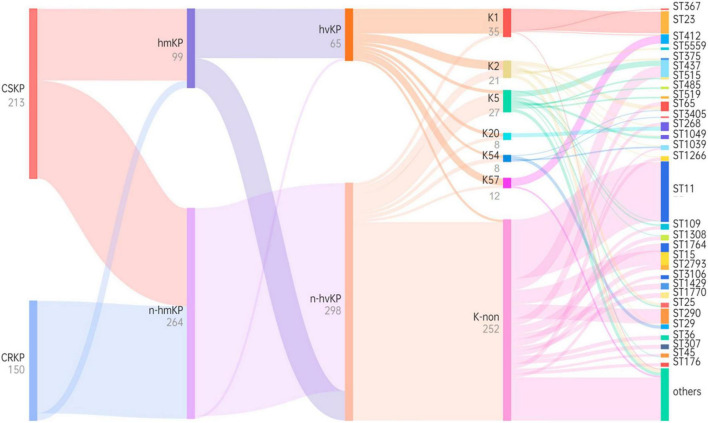
Phylogenetic tree based on virulence characteristics and ST typing. Black characters represent classifications, while gray characters represent quantities. The flow of bacterial strains is represented from **left** to **right**, and the thickness of the lines between nodes is proportional to the number of bacterial strains.

**FIGURE 6:**
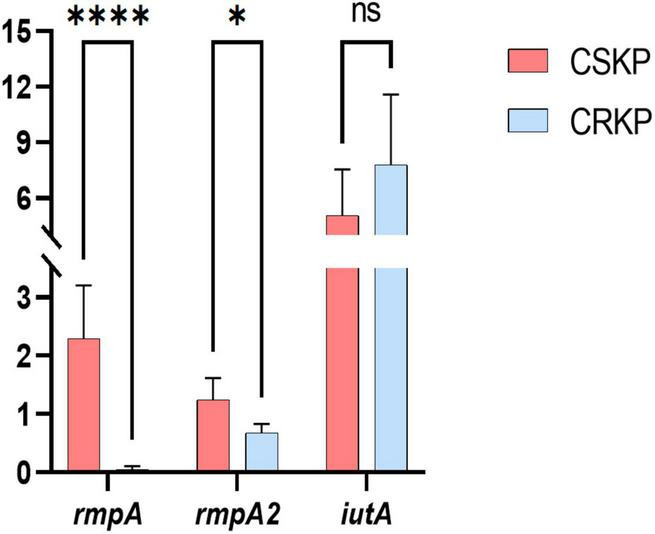
Differential gene expression efficiency of *rmpA*, *rmpA2*, and *iutA* in CSKP and CRKP. Using CSKP as a control, the differences in gene expression efficiency of CRKP in various genes are compared. ****P < 0.0001, *P < 0.05, and “ns” represents no statistically significant difference between the two groups.

**FIGURE 7:**
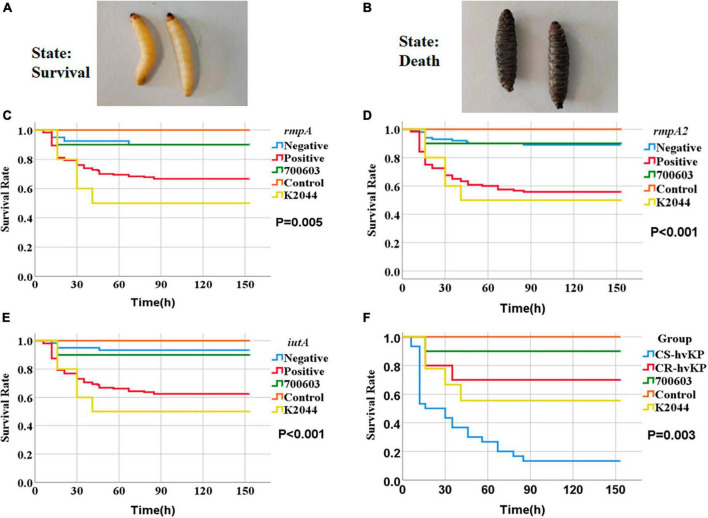
Survival curves of *Galleria mellonella* larvae in the infection model. The strains were derived from RT-qPCR consistently, as detailed in Supplementary Table 3. The criteria for determining larval mortality were blackening of the body and no response to touch. Panel **(A)** represents the living state of survival and panel **(B)** represents death. ATCC 700603 (shown as 700603), NTUH-K2044 (shown as K2044), and physiological saline control (shown as Control) in Panels **(C–E)** represent the survival rates of *Galleria mellonella* larvae at different time points with *rmpA*, *rmpA2*, and *iutA* gene-positive and gene-negative strains, respectively. The *P*-values indicate the significance between gene-positive and gene-negative strains in each case. Panel **(F)** shows the survival rates of *Galleria mellonella* larvae infected with CS-hvKP and CR-hvKP at different time points, with the *P*-value indicating the significance between the two groups.

### 2.8 Statistical analysis

In this study, we retrospectively analyzed the data of clinically isolated bacteria using WHONET 5.6 software. For data processing, we used SPSS 25.0 software (IBM, USA). The comparison of sample rates was analyzed using the Pearson’s chi-square 2 × 2 (2-sided) test or Fisher’s exact test (2-sided). Survival analysis was performed using the Log Rank (Mantel-Cox) test, with a *P*-value < 0.05 considered statistically significant. For the evaluation of virulence gene correlation, calculations, and graphical analyses were conducted using GraphPad Prism 9.5 software (GraphPad Software, San Diego, CA, USA).

## 3 Results

### 3.1 Strain source and specimen distribution

In this study, a total of 150 carbapenem-susceptible *Klebsiella pneumoniae* (CSKP) strains and 213 carbapenem-resistant *Klebsiella pneumoniae* (CRKP) strains were collected. The specimens comprised urine, sputum, bile, drainage fluid, puncture fluid, and blood. We found significant differences in the types of specimens between the two groups (detailed in Figure 1).

### 3.2 Detection of virulence genes and capsule genes

A total of 363 KP strains were examined for virulence genes. The positive rates of the 5 virulence genes (*rmpA*, *rmpA2*, *peg-344*, *iucA*, and *iroB*) in KP were 30.9, 34.2, 32.0, 41.9, and 27.3%, respectively. Further comparing the detection rates of virulence genes in carbapenem-susceptible KP (CSKP) and carbapenem-resistant KP (CRKP), we found that CSKP had significantly higher positive rates of genes *rmpA*, *peg-344*, and *iroB* compared to CRKP (*P* < 0.01), at rates of 44.1, 42.7, and 44.6%, respectively. However, there was no significant difference in the detection rates of genes *rmpA2* and *iucA* between the two groups (*P* > 0.05). Additionally, the detection rate of gene *ybts* in CSKP was 47.9%, significantly lower than that in CRKP (*P* < 0.01). Furthermore, CSKP and CRKP also showed significant differences in the detection rates of other virulence genes including *magA*, *aerobactin*, *alls*, *kfu*, and *entB* (*P* < 0.01) (Table 1).

**TABLE 1:** Detection rate of virulence and capsule genes.

Gene	CSKP	CRKP	*P*
	[*n* (%)]	[*n* (%)]	
*rmpA*	94 (44.1)	18 (12.0)	<0.001
*rmpA2*	74 (34.7)	50 (33.3)	0.781
*iucA*	95 (44.6)	57 (38.0)	0.209
*iroB*	97 (45.5)	2 (1.3)	<0.001
*peg-344*	91 (42.7)	25 (16.7)	<0.001
*iutA*	86 (40.4)	74 (49.3)	0.091
*magA*	32 (15.0)	0 (0)	<0.001
*aerobactin*	96 (45.1)	1 (0.7)	<0.001
*ybts*	102 (47.9)	113 (75.3)	<0.001
*alls*	44 (20.7)	2 (1.3)	<0.001
*rmkD*	185 (86.9)	137 (91.3)	0.184
*fimH*	200 (93.9)	138 (92.0)	0.482
*kfu*	81 (38.0)	27 (18.0)	<0.001
*entB*	206 (96.7)	134 (89.3)	0.004
*wabG*	203 (95.3)	140 (93.3)	0.417
K1	35 (16.4)	0 (0.0)	<0.001
K2	20 (9.4)	1 (0.7)	<0.001
K5	19 (8.9)	8 (5.3)	0.200
K20	7 (3.3)	1 (0.7)	0.147*
K54	8 (3.8)	0 (0.0)	0.023*
K57	10 (4.7)	2 (1.3)	0.133*
K non-typeable	114 (53.5)	138 (92.0)	<0.001

*Represents: the Fisher’s exact test (2-sided) is used.

For capsule genotypes, we found the number of K1, K2, K5, K20, K54, and K57 types were (35)9.6%, (21) 5.8%, (27)7.4%, (8) 2.2%, (8) 2.2%, and (12)3.3%, respectively. Furthermore, comparing the capsule genotypes between CSKP and CRKP, we found the detection rates of K1, K2, and K54 types in CSKP were significantly higher than in CRKP (Table 1).

We analyzed the distribution and correlation of virulence genes (Figure 2) and found that in CSKP, the genes *rmpA*, *rmpA2*, *iucA*, *iroB*, *peg-344*, and *aerobactin* tended to coexist. Additionally, the *iutA* gene showed a highly positive correlation with *rmpA*, *rmpA2*, *iucA*, *iroB*, and *aerobactin*. The *entB* gene showed a positive correlation with the *wabG* gene, and the K1 gene showed a positive correlation with the *magA* and *allS* genes. However, the K non-typeable gene showed a negative correlation with the *rmpA*, *iroB*, and *aerobactin* genes. In CRKP, the correlations among the genes were significantly reduced, the positive correlation was only found between the *iutA* and *iucA* genes, the *fimH* and *rmkD*, *wabG* genes, and between the *entB* and *wabG* genes.

### 3.3 Mucoid phenotype and virulent *Klebsiella pneumoniae*

Among the 363 strains of KP, 99 strains (27.3%) were identified as hypermucoviscous positive (hmKP). Among them, 90 strains (42.3%) in the CSKP group exhibited a high mucoviscosity phenotype, while only 9 strains (6.0%) in the CRKP group showed this phenotype, indicating a significant difference between the two groups (*P* < 0.01). We defined hvKP based on the detection of the *rmpA*, *rmpA2*, *peg-344*, *iucA*, and *iroB* genes. A total of 65 strains (17.9%) were determined as hvKP. Among them, the detection rate of CS-hvKP (30.0%) was significantly higher than that of CR-hvKP (0.7%) (Figure 3). At the same time, we divided the strains into sputum, blood, urine, and others sources (mostly sterile body fluids). The positive rate of hmKP was 22.2% in sputum, 36.2% in blood, 17.9% in urine and 35.7% in others isolates. The positive rate of blood isolates was similar to others isolates and higher than that of sputum and urine isolates. The detection of hvKP in each group also showed acacia results. The positive rate of hvKP was 13.5% in sputum, 21.3% in blood, 12.8% in urine and 25.0% in others isolates. The positive rate of blood isolates was similar to others isolates and higher than that of sputum and urine isolates (Figure 3).

### 3.4 MLST typing and correlation of virulence

Among the 363 strains of KP, the sequence type (ST) was successfully determined for 358. A total of 71 different ST types were detected. For CSKP, 58 different ST types were identified, with ST23 being the most common sequence type (*n* = 27, 12.7%), followed by ST65 (*n* = 11, 5.2%), ST268 (*n* = 10, 4.7%), and ST1764 (*n* = 10, 4.7%). On the other hand, for CRKP, 19 different ST types were identified, with ST11 being the most common sequence type (*n* = 75, 50.0%), followed by ST437 (*n* = 19, 15.2%) and ST268 (*n* = 10, 4.7%) (Figure 4).

Through examination of virulence characteristics and the sequence type (Figure 5), we observed that 98.5% of hvKP strains in this study were CSKP, while 1.5% were CRKP. Among hvKP strains, 65.65% were identified as hmKP. Additionally, the positive rate of capsule genes in hvKP was 95.38%, Among them, the detection rates of K1, K2, K5, K20, K54, and K57 were 47.69, 16.92, 6.15, 6.15, 4.62, and 13.85%, respectively.

Among hmKp isolates, the most common ST type was ST23 (*n* = 26, 26.3%), followed by ST412 (*n* = 7, 7.1%) and ST65 (*n* = 5, 5.1%). Among hvKP isolates, the most common ST type was also ST23 (*n* = 27, 41.5%), followed by ST412 (*n* = 7, 10.8%) and ST65 (*n* = 5, 7.7%). The ST types of these two groups showed a high degree of similarity. According to the classification of capsule serotypes, there were differences in the distribution of ST types among different capsule types. ST23 was primarily associated with K1 type (77.1%), while K2 type showed diversity, with ST65 being the predominant type (23.8%). K20 type was mainly associated with ST268 (62.5%) and ST368 (25.0%), while K54 type was mainly associated with ST29 (50.0%), and K57 type was primarily associated with ST412 (83.3%). More details can be found in Figure 5.

### 3.5 Relative expression levels of virulence genes

Based on the screening of virulence genes in this study, we selected genes *rmpA*, *rmpA2*, and *iutA*, which showed high positive rate in both CSKP and CRKP for RT-qPCR experiments (for detailed information on experimental strains, please refer to Supplementary Table 3). The results (Figure 6) showed significant differences in the expression of the same virulence genes among different strains. In CSKP, the expression level of the *rmpA* gene was significantly higher than that in CRKP (*P* < 0.01), followed by *rmpA2* (*P* = 0.015), while there was no significant difference in the expression of *iutA* (*P* = 0.200).

### 3.6 Pathogenicity test of KP strains using animal model

We studied the impact of different KP strains on larval survival rates in the model of *Galleria mellonella* larvae. Control strains included the negative control strain ATCC 700603, the positive control strain NTUH-K2044 (representing hvKP), and larvae injected with physiological saline. The survival rates of abovementioned controls were 90.0, 50.0, and 100% respectively. We also selected strains positive or negative for genes *rmpA*, *rmpA2*, and *iutA* for injection experiments (Supplementary Table 3). Compared to the control groups, we found significant differences in larval survival rates after injection with different strains expressing virulence genes. Particularly, the larval survival rate after injection with *rmpA*-positive strain was 66.7%, significantly lower than *rmpA*-negative strain (90.0%) (*P* = 0.005) (Figure 7C). The larval survival rate after injection with *rmpA2*-positive strain was 55.8%, significantly lower than *rmpA2*-negative strain (89.0%) (*P* < 0.001) (Figure 7D). The larval survival rate after injection with *iutA*-positive strain was 62.5%, significantly lower than *iutA*-negative strain (93.3%) (*P* < 0.001) (Figure 7E). In hvKP strains, the larval survival rate after injection with CS-hvKP strain (13.3%) was significantly lower than CR-hvKP strain (70.0%) (*P* = 0.003) (Figure 7F).

## 4 Discussion

*Klebsiella pneumoniae* can cause infections of the respiratory tract, bloodstream, urinary tract, gastrointestinal tract, liver and gallbladder, and central nervous system. The site of infection may vary depending on the virulence and migratory ability of the strain. In this study, we found that CSKP was mainly isolated from urine, while CRKP was mainly isolated from sputum samples. Additionally, there were statistically significant differences in the percentage of the two strains among other samples, indicating that these two types of strains may differ in terms of virulence and pathogenicity.

To date, a gold standard for defining hvKP is still lacking. HvKP has been previously determined based on the hypermucoviscous phenotype of colonies (Li et al., 2014; Zhan et al., 2017; Russo and Marr, 2019) due to its association with the synthesis of capsular polysaccharides. Capsular polysaccharides can enhance the survival and migratory ability of KP by inhibiting the host’s inflammatory response, leading to invasive infections. Our results showed that the detection rate of hypermucoviscous colonies in CSKP was 42.3%, while it was only 6.0% in CRKP. The positive rate of hvKP in blood (21.3%) was higher than that in sputum (13.5%) and urine (12.8%), indicating that blood isolates were more virulent than sputum and urine isolates. Soares de Moraes et al. (2022) proposed that *Klebsiella pneumoniae* of bloodstream infection was more virulent, and Li et al. (2023) found that the detection rate of virulence gene from blood isolates was higher than that of urine. These conclusions were consistent with our experimental results. It has been found that the virulence of KP is not solely dependent on its hypermucoviscosity (Zhang et al., 2015; Calfee, 2017). Our study showed that using hypermucoviscous as the criteria for hvKP resulted in a true positive rate of only 65.65%.

Previous work (Gu et al., 2018) indicated that pLVPK was an important virulence plasmid in hvKP, carrying various virulence genes. The absence of this plasmid reduces the virulence of hvKP strains. Therefore, researchers (Bialek-Davenet et al., 2014) have used genomic sequencing of the pLVPK plasmid to identify *rmpA*, *rmpA2*, *peg-344*, *iucA*, and *iroB* genes as molecular markers for hvKP, achieving an accuracy rate of over 95% (Russo and Marr, 2019). Among them, *rmpA* and *rmpA2* (Lin et al., 2020; Chen and Chen, 2021) are genes related to the regulation of mucoid phenotype and can regulate the synthesis and transcription of capsular polysaccharides, thickening the capsule and enhancing the virulence of the strain. *Peg-344* (Ye et al., 2016; Bulger et al., 2017) is a novel virulence factor that encodes an inner membrane transporter protein, the product of which is a permease in the superfamily of metabolite transporters. The *iucABCD* gene cluster encodes the aerobactin synthase, and the membrane protein receptor is encoded by the *iutA* gene. The *iroA* (*iroBCDN* gene cluster) encodes salmochelin (Paczosa and Mecsas, 2016; Palmer and Skaar, 2016; Nicolò et al., 2022), allowing bacteria to uptake sufficient iron for growth and reproduction. The results of this study showed that the positive rates of the *rmpA*, *peg-344*, and *iroB* genes in CSKP were 44.1, 42.7, and 45.5%, respectively, significantly higher than in CRKP, while there were no significant differences in the positive rates of the *rmpA2* and *iucA* genes. Moreover, the detection rates of genes such as *magA*, *aerobactin*, *alls*, *kfu*, and *entB* in CSKP were also higher than in CRKP. Capsule genes highly associated with hvKP included K1, K2, K5, K20, K54, and K57, and possess *rmpA*/*rmpA2* genes (Zhan et al., 2017; Russo et al., 2018; Yuan et al., 2019; Choby et al., 2020). This study found that in CSKP, the most common capsule types were K1, K2, and K5, while in CRKP, K5 was the main type, with significantly higher detection rates of K1, K2, and K54 in CSKP compared to CRKP.

By analyzing the correlation of virulence genes, we found that in CSKP, *rmpA*, *rmpA2*, *iucA*, *iroB*, *peg-344*, and *aerobactin* generally appeared together, and there was a high correlation among these virulence genes. However, in CRKP, the correlation between these genes was significantly reduced. This may be because these virulence genes participate in the expression of virulence and the pathogenic process, with the majority located on the same plasmids. Therefore, it can be speculated that the carriage of virulence plasmids may be more common in CSKP.

To further clarify the homogeneity and spread of CSKP and CRKP in the local area, MLST typing was performed. In CRKP strains, the globally epidemic ST types are ST258 and ST11 (Liao et al., 2020a; Su et al., 2020; Wei et al., 2022), but their spread is regional (Han et al., 2022). In this study, 19 ST types were detected in CRKP strains, with the most common ST type being ST11 (75, 50.0%), but ST258 was not detected. Different strains are restricted to their distinct clonal lineages (Yin et al., 2023). In China, ST11 is the most common clone of CRKP, with a rate of 80.7% (Pu et al., 2023), and a previous study revealed that the majority of CR-hvKP strains belonged to the ST11 type (Zhang et al., 2020), which was consistented with our results. In CSKP, a total of 58 ST types were detected, indicating significant diversity, but some epidemic strains were still observed. The most common ST type was ST23 (27, 12.7%). This is also the most common ST type in hvKP (Kocsis, 2023). Among the six common capsular serotype groups, we found that ST23 was mainly associated with K1 type (77.1%), while K2 type showed diversity and was mainly associated with ST65 (23.8%), K20 type was mainly associated with ST268 (62.5%) and ST368 (25.0%), K54 type was mainly associated with ST29 (50.0%), and K57 type was mainly associated with ST412 (83.3%), indicating clear clonal dissemination. This is consistent with the views of Kocsis (2023) and Li et al. (2023). Additionally, it was found that ST15, ST35, ST193, ST290, and ST412 types were detected in both CSKP and CRKP, which may be because the resistance of CRKP is mainly mediated by resistant plasmids, and as plasmids spread, it leads to differences in resistance among homologous strains. Similarly, transfer of virulence plasmids can occur between cKP and hvKP (Dong et al., 2019; Lam et al., 2019; Liao et al., 2020a; Han et al., 2022).

To further validate the virulence of CSKP and CRKP, we conducted RT-qPCR and a *Galleria mellonella* larvae infection model. Through RT-qPCR, we found that the relative expression level of the same virulence genes varied among different strains, with the most significant difference observed in *rmpA* gene between CSKP and CRKP (*P* < 0.001), followed by *rmpA2* (*P* = 0.015). In addition, Zhu et al. (2021b) constructed *blaKPC-2* transformants by expressing *blaKPC-2* in hvKP through *in vitro* transformation. In the *blaKPC-2* transformants, the relative expression levels of *rmpA* and *rmpA2* genes were approximately 50% of those in the wild-type strain, indicating that the expression level of virulence genes decreases after acquiring resistance. This may be related to the energy burden of bacteria, since the expression of resistant genes consumes energy, resulting in a reduction in the expression of virulence genes (Drancourt et al., 2001). Fursova et al. (2021) studied the bacterial resistance gene expression of multidrug-resistant KP (MDR-KP) and multidrug-resistant hvKP (MDR-hvKP) and found that the β-lactamase and carbapenemase genes of the former had higher expression levels, which also indicated that MDR-hvKP had a higher metabolic load due to multiple resistance and virulence factors (Mendes et al., 2023). Zhang Y. et al. (2021) studied the suitability of MDR-hvKPstrains after resistance plasmid and resistant plasmid, and found that the stability of resistant plasmid was higher. The results of the *Galleria mellonella* larvae infection model also supported this notion. We found that the survival rate of larvae infected with CS-hvKP strains was lower than that of larvae infected with CR-hvKP strains. Furthermore, the experimental results of the *Galleria mellonella* larvae infection model also revealed that the survival rate of larvae infected with strains positive for *rmpA*, *rmpA2*, and *iutA* genes was significantly lower than that of larvae infected with strains negative for these genes. This further indicates that these genes play an important role in the expression of virulence of *Klebsiella pneumoniae*.

This study did not carry out drug resistance gene detection, could not combine carbapenem resistance phenotype with genes, and could not reflect the correlation between virulence genes and drug resistance genes, which has certain limitations. Further research will be carried out in the future.

## 5 Conclusion

In general, among clinically isolated *Klebsiella pneumoniae*, hmKP is more manifested as hvKP, which needs more attention. CSKP carries more virulence genes than CRKP, and the distribution of these genes in CSKP is relatively balanced. Most hvKP strains belong to CSKP, while there are fewer in CRKP. CRKP strains show significant homogeneity, with the predominant sequence type being ST11; whereas CSKP strains exhibit relative diversity, but there is still some evidence of clonal dissemination, with ST23 being the common type. There may be differences in virulence gene expression between hv-CSKP and hv-CRKP, and in the *Galleria mellonella* larvae model, the survival rate of hv-CSKP-infected larvae is lower than that of hv-CRKP-infected larvae.

## Data availability statement

The original contributions presented in this study are included in the article/Supplementary material, further inquiries can be directed to the corresponding authors.

## Ethics statement

The manuscript presents research on animals that do not require Ethical approval for their study.

## Author contributions

MJ: Data curation, Formal analysis, Investigation, Methodology, Writing – original draft. XQ: Investigation, Project administration, Supervision, Writing – original draft. SS: Conceptualization, Data curation, Investigation, Writing – original draft. RZ: Methodology, Writing – original draft. WL: Data curation, Investigation, Writing – original draft. CL: Writing – review and editing. YT: Funding acquisition, Resources, Writing – review and editing. YW: Funding acquisition, Resources, Writing – review and editing. QL: Methodology, Supervision, Writing – review and editing. QW: Funding acquisition, Resources, Writing – review and editing.
