# Supplementary material for: Differences in molecular characteristics and expression of virulence genes in carbapenem-resistant and sensitive Klebsiella pneumoniae isolates in Ningbo, China
Source: Front Microbiol. 2024 Feb 7;15:1356229. doi: 10.3389/fmicb.2024.1356229 (PMC10881320; doi:10.3389/fmicb.2024.1356229)
Supplement: Supplementary file 1 [file Data_Sheet_1.doc]

**Supplemental material**

**Supplementary Table1 Drug susceptibility test**

| **Medicine** |  | **CSKP(n=213)** | |  | **CRKP(n=150)** | |
| --- | --- | --- | --- | --- | --- | --- |
|  |  | **n** | **（%）** |  | **n** | **（%）** |
| Ampicillin/sulbactam |  | 74 | 34.7 |  | 150 | 100 |
| ceftriaxone |  | 61 | 28.6 |  | 148 | 98.7 |
| furantoin |  | 54 | 25.4 |  | 138 | 92.0 |
| Cotrimoxazole |  | 53 | 24.9 |  | 84 | 56.0 |
| aztreonam |  | 43 | 20.2 |  | 144 | 96.0 |
| gentamicin |  | 35 | 16.4 |  | 125 | 83.3 |
| ceftazidime |  | 33 | 15.5 |  | 146 | 97.3 |
| Ciprofloxacin |  | 33 | 15.5 |  | 141 | 94.0 |
| Levofloxacin |  | 24 | 11.3 |  | 141 | 94.0 |
| cefepime |  | 23 | 10.8 |  | 144 | 96.0 |
| Cefoperazone/Sulbactam |  | 17 | 8.0 |  | 146 | 97.3 |
| tobramycin |  | 16 | 7.5 |  | 116 | 77.3 |
| Piperacillin/Tazobactam |  | 8 | 3.8 |  | 135 | 90.0 |
| amikacin |  | 4 | 1.9 |  | 98 | 65.3 |
| Ertapenem |  | 0 | 0 |  | 149 | 99.3 |
| cefotetan |  | 1 | 0.5 |  | 135 | 90.0 |
| Imipenem |  | 0 | 0 |  | 150 | 100 |

**Supplementary Table 2** Sequences of the pcr primers

| **Gene** | **Primer sequences**  **(5’-3’)** | **Product**  **（bp）** | **Annealing**  **（℃）** | **References** |
| --- | --- | --- | --- | --- |
| *rmpA* | F: ACTGGGCTACCTCTGCTTCA | 516 | 55 | (Lin et al., 2020) |
|  | R: CTTGCATGAGCCATCTTTCA |  |  |  |
| *rmpA2* | F: CTTTATGTGCAATAAGGATGTT | 451 | 52 | (Zhang et al., 2022) |
|  | R: CCTCCTGGAGAGTAAGCATT |  |  |  |
| *iucA* | F：AATCAATGGCTATTCCCGCTG | 239 | 59 | (Du et al., 2022) |
|  | R：CGCTTCACTTCTTTCACTGACAGG |  |  |  |
| *iroB* | F：ATCTCATCATCTACCCTCCGCTC | 235 | 58 | (Zhang et al., 2022) |
|  | R：GGTTCGCCGTCGTTTTCAA |  |  |  |
| *peg-344* | F：CTTGAAACTATCCCTCCAGTC | 508 | 56 | (Du et al., 2022) |
|  | R：CCAGCGAAAGAATAACCCC |  |  |  |
| *iutA* | F: ACCTGGGTTATCGAAAACGC | 1115 | 55 | (Liu et al., 2019) |
|  | R: GATGTCATAGCCTGATTGC |  |  |  |
| *magA* | F: GGTGCTCTTTACATCATTGC | 1283 | 53 | (Lin et al., 2020) |
|  | R: GCAATGGCCATTTGCGTTAG |  |  |  |
| *aerobactin* | F: GCATAGGCGGATACGAACAT | 556 | 53 | (Zhou et al., 2020) |
|  | R: CACAGGGCAATTGCTTACCT |  |  |  |
| *ybtS* | F: GACGGAAACAGCACGGTAAA | 242 | 50 | (Zhou et al., 2020) |
|  | R: GAGCATAATAAGGCGAAAGA |  |  |  |
| *alls* | F: CATTACGCACCTTTGTCAGC | 764 | 57 | (Zhou et al., 2020) |
|  | R: GAATGTGTCGGCGATCAGCTT |  |  |  |
| *mrkD* | F: CCACCAACTATTCCCTCGAA | 226 | 54 | (Dan et al., 2023) |
|  | R: ATGGAACCCACATCGACATT |  |  |  |
| *fimH* | F: ATGAACGCCTGGTCCTTTGC | 688 | 56 | (Kot et al., 2023) |
|  | R: GCTGAACGCCTATCCCCTGC |  |  |  |
| *kfu* | F: GAAGTGACGCTGTTTCTGGC | 797 | 58 | (Li et al., 2023) |
|  | R: TTTCGTGTGGCCAGTGACTC |  |  |  |
| *entB* | F: GTCAACTGGGCCTTTGAGCCGGTC | 400 | 59 | (Zhou et al., 2020) |
|  | R: TATGGGCGTAAACGCCGGTGAT |  |  |  |
| *wabG* | F: ACCATCGGCCATTTGATAGA | 683 | 55 | (Candan and Aksöz, 2015) |
|  | R: CGGACTGGCAGATCCATATC |  |  |  |
| K1 | F: GGTGCTCTTTACATCATTGC | 1283 | 55 | (Liu et al., 2023) |
|  | R: GCCCAGGTTAATGAATCCGT |  |  |  |
| K2 | F: GACCCGATATTCATACTTGACAGAG | 641 | 53 | (Zhou et al., 2020) |
|  | R: CCTGAAGTAAAATCGTAAATAGAT |  |  |  |
| K5 | F: GCCACCTCTAAGCATATAGC | 540 | 55 | (Liu et al., 2023) |
|  | R: CGCACCAGTAATTCCAACAG |  |  |  |
| K20 | F: CGGTGCTACAGTGCATCATT | 741 | 56 | (Liu et al., 2023) |
|  | R: GTTATACGATGCTCAGTCGC |  |  |  |
| K54 | F: CATTAGCTCAGTGGTTGGCT | 881 | 55 | (Liu et al., 2023) |
|  | R: GCTTGACAAACACCATAGCAG |  |  |  |
| K57 | F: CGACAAATCTCTCCTGACGA | 1037 | 50 | (Liu et al., 2023) |
|  | R: CGCGACAAACATAACACTCG |  |  |  |
| *gap A* | F: TGAAATATGACTCCACTCACGG | 662 | 59 | (Lin et al., 2020) |
|  | R: CTTCAGAAGCGGCTTTGATGGCTT |  |  |  |
| *inf B* | F: CTCGCTGCTGGACTATATTCG | 462 | 56 | (Lin et al., 2020) |
|  | R: CGCTTTCAGCTCAAGAACTTC |  |  |  |
| *rpoB* | F: GGCGAAATGGCWGAGAACCA | 1075 | 56 | (Lin et al., 2020) |
|  | R: GAGTCTTCGAAGTTGTAACC |  |  |  |
| *mdh* | F：F: CCCAACTCGCTTCAGGTTCAG | 756 | 59 | (Lin et al., 2020) |
|  | R: CCGTTTTTCCCCAGCAGCAG |  |  |  |
| *pgi* | F: GAGAAAAACCTGCCTGTACTGCTGGC | 566 | 59 | (Lin et al., 2020) |
|  | R: CGCGCCACGCTTTATAGCGGTTAAT |  |  |  |
| *phoE* | F: ACCTACCGCAACACCGACTTCTTCGG | 602 | 59 | (Lin et al., 2020) |
| *tonB* | R: TGATCAGAACTGGTAGGTGAT  F: CTTTATACCTCGGTACATCAGGTT  R: ATTCGCCGGCTGRGCRGAGAG | 539 | 59 | (Lin et al., 2020) |
| *q-rmpA* | F: AGGGAAATGGGGAGGGTACAAAATG | 78 | 60 | This studay |
|  | R: CCCGAAACGTCAAGCCACATC |  |  |  |
| *q-rmpA2* | F: AGTCAATGGATGTGGCTTGAC | 71 | 57 | This studay |
|  | R: ATGTCATAATCACACCCTTGAGG: |  |  |  |
| *q-iutA* | F: ACATCCGCCGACGCCATTC | 120 | 62 | This studay |
|  | R: CACGCCCTGGGAGAAGTTGAG |  |  |  |
| *16SRNA* | F: AGAGTTTGATCTTGGCTCAG | - | 55 | This studay |
|  | R: GGTTACCTTGTTACGACTT |  |  |  |

**References**

Candan, E.D., and Aksöz, N. (2015). *Klebsiella pneumoniae*: characteristics of carbapenem resistance and virulence factors. *Acta Biochim Pol* 62**,** 867-874. doi:10.18388/abp.2015_1148.

Dan, B., Dai, H., Zhou, D., Tong, H., and Zhu, M. (2023). Relationship Between Drug Resistance Characteristics and Biofilm Formation in *Klebsiella Pneumoniae* Strains. *Infect Drug Resist* 16**,** 985-998. doi:10.2147/idr.S396609.

Du, Q., Pan, F., Wang, C., Yu, F., Shi, Y., Liu, W., et al. (2022). Nosocomial dissemination of hypervirulent *Klebsiella pneumoniae* with high-risk clones among children in Shanghai. *Front Cell Infect Microbiol* 12**,** 984180. doi:10.3389/fcimb.2022.984180.

Kot, B., Piechota, M., Szweda, P., Mitrus, J., Wicha, J., Grużewska, A., et al. (2023). Virulence analysis and antibiotic resistance of *Klebsiella pneumoniae* isolates from hospitalised patients in Poland. *Sci Rep* 13**,** 4448. doi:10.1038/s41598-023-31086-w.

Li, H.F., Zhang, L.X., Zhang, W.L., Li, J., Li, Y.Q., and Hu, T.P. (2023). Study on Virulence Genes, Drug Resistance and Molecular Epidemiology of *Klebsiella pneumoniae* with High Virulence in Inner Mongolia, China. *Infect Drug Resist* 16**,** 1133-1144. doi:10.2147/idr.S391468.

Lin, Z.W., Zheng, J.X., Bai, B., Xu, G.J., Lin, F.J., Chen, Z., et al. (2020). Characteristics of Hypervirulent *Klebsiella pneumoniae*: Does Low Expression of rmpA Contribute to the Absence of Hypervirulence? *Front Microbiol* 11**,** 436. doi:10.3389/fmicb.2020.00436.

Liu, P., Yang, A., Tang, B., Wang, Z., Jian, Z., Liu, Y., et al. (2023). Molecular epidemiology and clinical characteristics of the type VI secretion system in *Klebsiella pneumoniae* causing abscesses. *Front Microbiol* 14**,** 1181701. doi:10.3389/fmicb.2023.1181701.

Liu, Y., Du, F.L., Xiang, T.X., Wan, L.G., Wei, D.D., Cao, X.W., et al. (2019). High Prevalence of Plasmid-Mediated Quinolone Resistance Determinants Among Serotype K1 Hypervirulent *Klebsiella pneumoniae* Isolates in China. *Microb Drug Resist* 25**,** 681-689. doi:10.1089/mdr.2018.0173.

Zhang, Y., Xu, Y., and Huang, Y. (2022). Virulence Genotype and Correlation of Clinical Severeness with Presence of the Type VI Secretion System in *Klebsiella pneumoniae* Isolates Causing Bloodstream Infections. *Infect Drug Resist* 15**,** 1487-1497. doi:10.2147/idr.S353858.

Zhou, M., Lan, Y., Wang, S., Liu, Q., Jian, Z., Li, Y., et al. (2020). Epidemiology and molecular characteristics of the type VI secretion system in *Klebsiella pneumoniae* isolated from bloodstream infections. *J Clin Lab Anal* 34**,** e23459. doi:10.1002/jcla.23459.

**Supplementary Table 3** List of strains selected for RT-qpcr and the *Galleria mellonella* larvae model

| **Group** | **Strain** | **MLST** | **Capsulp** | ***iutA*** | ***rmpA*** | ***rmpA2*** | ***iucA*** | ***iroB*** | ***peg-344*** | ***magA*** | ***areobactin*** | ***ybts*** | ***alls*** | ***rmkD*** | ***fimH*** | ***kfu*** | ***entB*** | ***wabG*** |
| --- | --- | --- | --- | --- | --- | --- | --- | --- | --- | --- | --- | --- | --- | --- | --- | --- | --- | --- |
| CSKP | 27 | ST412 | K57 | **+** | **+** | **+** | **+** | **+** | **+** | **-** | **+** | **-** | **-** | **+** | **+** | **-** | **+** | **+** |
| CSKP | 113 | ST412 | K57 | **+** | **+** | **+** | **+** | **+** | **+** | **-** | **+** | **-** | **-** | **+** | **+** | **-** | **+** | **+** |
| CSKP | 142 | ST5107 | K57 | **+** | **+** | **+** | **+** | **+** | **+** | **-** | **+** | **+** | **-** | **+** | **+** | **-** | **+** | **+** |
| CRKP | 411 | ST412 | K57 | **+** | **+** | **+** | **+** | **+** | **+** | **-** | **+** | **+** | **-** | **+** | **+** | **-** | **+** | **+** |
| CSKP | 31 | ST368 | K20 | **+** | **+** | **+** | **+** | **-** | **+** | **-** | **+** | **+** | **-** | **+** | **+** | **-** | **+** | **+** |
| CSKP | 174 | ST1049 | K5 | **+** | **+** | **+** | **+** | **+** | **-** | **-** | **+** | **+** | **-** | **+** | **+** | **+** | **+** | **+** |
| CRKP | 385 | ST11 | K non-typable | **+** | **+** | **+** | **+** | **-** | **-** | **-** | **-** | **-** | **-** | **+** | **+** | **-** | **+** | **+** |
| CRKP | 431 | ST11 | K non-typable | **+** | **+** | **+** | **+** | **-** | **-** | **-** | **-** | **+** | **-** | **+** | **+** | **-** | **+** | **+** |
| CSKP | 157 | ST25 | K2 | **+** | **+** | **-** | **+** | **+** | **-** | **-** | **+** | **+** | **-** | **+** | **+** | **-** | **+** | **+** |
| CSKP | 171 | ST65 | K54 | **+** | **+** | **-** | **+** | **+** | **+** | **-** | **+** | **+** | **-** | **+** | **+** | **-** | **+** | **+** |
| CRKP | 343 | ST437 | K non-typable | **+** | **+** | **-** | **+** | **-** | **-** | **-** | **-** | **-** | **-** | **+** | **+** | **-** | **+** | **+** |
| CRKP | 344 | ST437 | K non-typable | **+** | **+** | **-** | **+** | **-** | **-** | **-** | **-** | **-** | **-** | **+** | **+** | **-** | **+** | **+** |
| CSKP | 12 | ST412 | K57 | **+** | **-** | **+** | **+** | **+** | **+** | **-** | **+** | **-** | **-** | **+** | **+** | **-** | **+** | **+** |
| CSKP | 36 | ST793 | K1 | **+** | **-** | **+** | **+** | **+** | **+** | **-** | **+** | **+** | **+** | **+** | **+** | **+** | **+** | **+** |
| CRKP | 384 | ST15 | K non-typable | **+** | **-** | **+** | **+** | **-** | **+** | **-** | **-** | **-** | **-** | **+** | **+** | **+** | **+** | **+** |
| CRKP | 412 | ST11 | K non-typable | **+** | **-** | **+** | **+** | **-** | **+** | **-** | **-** | **+** | **-** | **+** | **+** | **-** | **+** | **+** |
| CSKP | 198 | ST485 | K5 | **-** | **+** | **+** | **+** | **+** | **+** | **-** | **+** | **+** | **-** | **+** | **+** | **-** | **+** | **+** |
| CSKP | 211 | ST344 | K non-typable | **-** | **+** | **-** | **-** | **+** | **+** | **-** | **+** | **+** | **-** | **-** | **+** | **+** | **+** | **+** |
| CSKP | 214 | ST1049 | K5 | **-** | **+** | **-** | **+** | **+** | **+** | **-** | **+** | **+** | **-** | **+** | **+** | **+** | **+** | **+** |
| CRKP | 361 | ST437 | K non-typable | **-** | **+** | **-** | **-** | **-** | **+** | **-** | **-** | **-** | **-** | **+** | **+** | **-** | **+** | **+** |
| CRKP | 369 | ST11 | K non-typable | **-** | **+** | **-** | **-** | **-** | **-** | **-** | **-** | **+** | **-** | **+** | **-** | **-** | **+** | **+** |
| CRKP | 376 | ST11 | K non-typable | **-** | **+** | **-** | **-** | **-** | **-** | **-** | **-** | **+** | **-** | **+** | **+** | **-** | **+** | **+** |

27, 113, 142, 411all carry *iutA*, *rmpA*, *rmpA2*, *iucA*, *iroB*, *peg-344*, *areobactin*, *rmkD*, *fimH*, *entB*, *wabG*. 31, 174, 385, 431 all carried *iutA*, *rmpA*, *rmpA2*, *iucA*, *rmkD*, *fimH*, *entB*, *wabG*. 157, 171, 343, 344 did not express *rmpA2*, but carry *iutA*, *rmpA*, *iucA*, *rmkD*, *fimH*, *entB*, *wabG*. 12, 36, 384, 412 do not express *rmpA*, but carry *iutA*, *rmpA2*, *iucA*, *peg-344*, *rmkD*, *fimH*, *entB*, *wabG*. 198, 211, 214, 361, 369, 376 did not express *iutA*, but expressed *rmpA*, *entB* and *wabG*
