# Supplementary material for: Differences in molecular characteristics and expression of virulence genes in carbapenem-resistant and sensitive Klebsiella pneumoniae isolates in Ningbo, China
Source: Front Microbiol. 2024 Feb 7;15:1356229. doi: 10.3389/fmicb.2024.1356229 (PMC10881320; doi:10.3389/fmicb.2024.1356229)
Supplement: Supplementary file 2 [file Data_Sheet_2.docx]

**Manuscript data summary**

**Data 1**  Source of clinical isolates

| **Style** | **CSKP**  **[n（%）]** | **CRKP**  **[n（%）]** | **Chi-square test** | |
| --- | --- | --- | --- | --- |
|  |  |  | ***χ^2^*** | ***P*** |
| Sputum | 41（19.2） | 85（56.7） | 54.377 | <0.001 |
| Blood | 30（14.1） | 17（11.3） | 0.591 | 0.442 |
| Urine | 55（25.8） | 23（15.3） | 5.739 | 0.017 |
| Bile | 25（11.7） | 2（1.3） | 13.837 | <0.001 |
| Hydrothorax/ascite | 6（2.8） | 1（0.7） | - | 0.247* |
| Secreta | 9（4.2） | 13（8.7） | 3.049 | 0.081 |
| Drainage fluid | 20（9.4） | 4（2.7） | 6.443 | 0.011 |
| Puncture fluid | 14（6.6） | 0（0） | 10.255 | 0.001 |
| Faeces | 0（0） | 2（1.3） | - | 0.170* |
| Conduit | 2（0.9） | 1（0.7） | - | >0.999* |
| Lavage fluid | 3（1.4） | 1（0.7） | - | 0.645* |
| Cerebrospinal fluid | 2(0.9) | 0(0) | - | 0.514* |
| Gastric fluid | 1(0.5) | 0(0) | - | >0.999* |
| Others | 5(2.3) | 1(0.7) | - | 0.407* |

Note: "*" represents: Fisher’s exact test (2-sided) is used.

In manuscript Figure 1, "Faeces" and "Gastric fluid" were classified into "Others" because there were too many types of specimens and no labels could be put down

**Date2** String test of CSKP and CRKP

| **Group** | **Count**  **（n）** | **hmKP**  **[n（%）]** | **n-hmKP**  **[n（%）]** | **Chi-square test** | |
| --- | --- | --- | --- | --- | --- |
|  |  |  |  | ***χ^2^*** | ***P*** |
| CSKP | 213 | 90(42.3) | 123(57.7) | 58.323 | <0.001 |
| CRKP | 150 | 9(6.0) | 141(94.0) |  |  |

**Date3** String test of different specimen types

| **Types** | **hmKP**  **(n)** | **n-hmKP**  **(n)** | **positive rate**  **(%)** |
| --- | --- | --- | --- |
| \| Sputum \| \| --- \| | 28 | 98 | 22.2 |
| Blood | 17 | 30 | 36.2 |
| Urine | 14 | 64 | 17.9 |
| Others | 40 | 72 | 35.7 |

**Date4**  Hvkp strains of CSKP and CRKP

| **Group** | **Count**  **（n）** | **hvKP**  **[n（%）]** | **n-hvKP**  **[n（%）]** | **Chi-square test** | |
| --- | --- | --- | --- | --- | --- |
|  |  |  |  | ***χ^2^*** | ***P*** |
| CSKP | 213 | 64(30.0) | 149(70.0) | 51.684 | <0.001 |
| CRKP | 150 | 1(0.7) | 149(99.3) |  |  |

**Date5** Hvkp strains of different specimen types

| **Types** | **hvKP**  **(n)** | **n-hvKP**  **(n)** | **positive rate**  **(%)** |
| --- | --- | --- | --- |
| \| Sputum \| \| --- \| | 17 | 109 | 13.5 |
| Blood | 10 | 37 | 21.3 |
| Urine | 10 | 68 | 12.8 |
| Others | 28 | 84 | 25.0 |

**Date6** Pearson correlation coefficients of genes in CSKP

| **Gene** | ***rmpA*** | ***rmpA2*** | ***iucA*** | ***iroB*** | ***peg-344*** | ***iutA*** | ***magA*** | ***aerobactin*** | ***ybts*** | ***alls*** | ***rmkD*** | ***fim H*** |
| --- | --- | --- | --- | --- | --- | --- | --- | --- | --- | --- | --- | --- |
| ***rmpA*** | 1.00 | 0.76 | 0.74 | 0.95 | 0.90 | 0.71 | 0.47 | 0.96 | 0.49 | 0.29 | 0.26 | 0.19 |
| ***rmpA2*** | 0.76 | 1.00 | 0.71 | 0.78 | 0.79 | 0.73 | 0.49 | 0.81 | 0.37 | 0.41 | 0.28 | 0.19 |
| ***iucA*** | 0.74 | 0.71 | 1.00 | 0.75 | 0.70 | 0.92 | 0.47 | 0.74 | 0.46 | 0.31 | 0.32 | 0.15 |
| ***iroB*** | 0.95 | 0.78 | 0.75 | 1.00 | 0.89 | 0.73 | 0.46 | 0.97 | 0.48 | 0.30 | 0.24 | 0.15 |
| ***peg-344*** | 0.90 | 0.79 | 0.70 | 0.89 | 1.00 | 0.66 | 0.46 | 0.90 | 0.43 | 0.33 | 0.25 | 0.18 |
| ***iutA*** | 0.71 | 0.73 | 0.92 | 0.73 | 0.66 | 1.00 | 0.48 | 0.72 | 0.42 | 0.36 | 0.29 | 0.17 |
| ***magA*** | 0.47 | 0.49 | 0.47 | 0.46 | 0.46 | 0.48 | 1.00 | 0.46 | 0.36 | 0.66 | 0.16 | 0.05 |
| ***aerobactin*** | 0.96 | 0.81 | 0.74 | 0.97 | 0.90 | 0.72 | 0.46 | 1.00 | 0.49 | 0.31 | 0.27 | 0.19 |
| ***ybtS*** | 0.49 | 0.37 | 0.46 | 0.48 | 0.43 | 0.42 | 0.36 | 0.49 | 1.00 | 0.21 | 0.09 | 0.09 |
| ***alls*** | 0.29 | 0.41 | 0.31 | 0.30 | 0.33 | 0.36 | 0.66 | 0.31 | 0.21 | 1.00 | 0.06 | 0.08 |
| ***rmkD*** | 0.26 | 0.28 | 0.32 | 0.24 | 0.25 | 0.29 | 0.16 | 0.27 | 0.09 | 0.06 | 1.00 | 0.54 |
| ***fim H*** | 0.19 | 0.19 | 0.15 | 0.15 | 0.18 | 0.17 | 0.05 | 0.19 | 0.09 | 0.08 | 0.54 | 1.00 |
| ***kfu*** | 0.12 | 0.08 | 0.19 | 0.12 | 0.13 | 0.20 | 0.46 | 0.13 | 0.20 | 0.39 | -0.12 | 0.12 |
| ***entB*** | 0.16 | 0.13 | 0.11 | 0.12 | 0.16 | 0.10 | 0.08 | 0.17 | 0.07 | 0.09 | 0.47 | 0.72 |
| ***wab G*** | 0.15 | 0.12 | 0.15 | 0.11 | 0.15 | 0.14 | 0.09 | 0.16 | 0.04 | 0.11 | 0.50 | 0.78 |
| **K1** | 0.47 | 0.53 | 0.49 | 0.48 | 0.49 | 0.51 | 0.95 | 0.49 | 0.36 | 0.71 | 0.17 | 0.06 |
| **K2** | 0.36 | 0.17 | 0.23 | 0.35 | 0.28 | 0.26 | -0.14 | 0.36 | 0.14 | -0.16 | 0.08 | 0.08 |
| **K5** | 0.02 | -0.02 | 0.02 | 0.04 | -0.07 | -0.02 | -0.13 | 0.01 | 0.06 | -0.16 | -0.02 | -0.06 |
| **K20** | 0.15 | 0.20 | 0.10 | 0.10 | 0.16 | 0.06 | -0.08 | 0.15 | 0.03 | -0.03 | 0.07 | 0.05 |
| **K54** | 0.02 | 0.01 | 0.12 | 0.02 | 0.03 | 0.14 | -0.08 | 0.02 | 0.11 | -0.10 | 0.08 | 0.05 |
| **K57** | 0.21 | 0.26 | 0.20 | 0.24 | 0.26 | 0.18 | -0.09 | 0.25 | -0.08 | -0.11 | 0.09 | 0.06 |
| **K non-typeable** | -0.73 | -0.66 | -0.68 | -0.74 | -0.66 | -0.67 | -0.45 | -0.75 | -0.41 | -0.25 | -0.25 | -0.12 |

**Continued form Date6** Pearson correlation coefficients of genes in CSKP

| **Gene** | ***kfu*** | ***entB*** | ***wab G*** | **K1** | **K2** | **K5** | **K20** | **K54** | **K57** | **K non-typeable** |
| --- | --- | --- | --- | --- | --- | --- | --- | --- | --- | --- |
| ***rmpA*** | 0.12 | 0.16 | 0.15 | 0.47 | 0.36 | 0.02 | 0.15 | 0.02 | 0.21 | -0.73 |
| ***rmpA2*** | 0.08 | 0.13 | 0.12 | 0.53 | 0.17 | -0.02 | 0.20 | 0.01 | 0.26 | -0.66 |
| ***iucA*** | 0.19 | 0.11 | 0.15 | 0.49 | 0.23 | 0.02 | 0.10 | 0.12 | 0.20 | -0.68 |
| ***iroB*** | 0.12 | 0.12 | 0.11 | 0.48 | 0.35 | 0.04 | 0.10 | 0.02 | 0.24 | -0.74 |
| ***peg-344*** | 0.13 | 0.16 | 0.15 | 0.49 | 0.28 | -0.07 | 0.16 | 0.03 | 0.26 | -0.66 |
| ***iutA*** | 0.20 | 0.10 | 0.14 | 0.51 | 0.26 | -0.02 | 0.06 | 0.14 | 0.18 | -0.67 |
| ***magA*** | 0.46 | 0.08 | 0.09 | 0.95 | -0.14 | -0.13 | -0.08 | -0.08 | -0.09 | -0.45 |
| ***aerobactin*** | 0.13 | 0.17 | 0.16 | 0.49 | 0.36 | 0.01 | 0.15 | 0.02 | 0.25 | -0.75 |
| ***ybtS*** | 0.20 | 0.07 | 0.04 | 0.36 | 0.14 | 0.06 | 0.03 | 0.11 | -0.08 | -0.41 |
| ***alls*** | 0.39 | 0.09 | 0.11 | 0.71 | -0.16 | -0.16 | -0.03 | -0.10 | -0.11 | -0.25 |
| ***rmkD*** | -0.12 | 0.47 | 0.50 | 0.17 | 0.08 | -0.02 | 0.07 | 0.08 | 0.09 | -0.25 |
| ***fim H*** | 0.12 | 0.72 | 0.78 | 0.06 | 0.08 | -0.06 | 0.05 | 0.05 | 0.06 | -0.12 |
| ***kfu*** | 1.00 | 0.14 | 0.17 | 0.49 | -0.12 | 0.03 | -0.14 | -0.10 | -0.17 | -0.14 |
| ***entB*** | 0.14 | 1.00 | 0.83 | 0.08 | 0.06 | -0.13 | 0.03 | 0.04 | 0.04 | -0.07 |
| ***wab G*** | 0.17 | 0.83 | 1.00 | 0.10 | 0.00 | -0.09 | 0.04 | 0.04 | 0.05 | -0.07 |
| **K1** | 0.49 | 0.08 | 0.10 | 1.00 | -0.14 | -0.14 | -0.08 | -0.09 | -0.10 | -0.48 |
| **K2** | -0.12 | 0.06 | 0.00 | -0.14 | 1.00 | -0.10 | -0.06 | -0.06 | -0.07 | -0.35 |
| **K5** | 0.03 | -0.13 | -0.09 | -0.14 | -0.10 | 1.00 | -0.06 | -0.06 | -0.07 | -0.34 |
| **K20** | -0.14 | 0.03 | 0.04 | -0.08 | -0.06 | -0.06 | 1.00 | -0.04 | -0.04 | -0.20 |
| **K54** | -0.10 | 0.04 | 0.04 | -0.09 | -0.06 | -0.06 | -0.04 | 1.00 | -0.04 | -0.21 |
| **K57** | -0.17 | 0.04 | 0.05 | -0.10 | -0.07 | -0.07 | -0.04 | -0.04 | 1.00 | -0.24 |
| **K non-typeable** | -0.14 | -0.07 | -0.07 | -0.48 | -0.35 | -0.34 | -0.20 | -0.21 | -0.24 | 1.00 |

**Date7** Pearson correlation coefficients of genes in CRKP

| **Gene** | ***rmpA*** | ***rmpA2*** | ***iucA*** | ***iroB*** | ***peg-344*** | ***iutA*** | ***magA*** | ***aerobactin*** | ***ybts*** | ***alls*** | ***rmkD*** | ***fim H*** |
| --- | --- | --- | --- | --- | --- | --- | --- | --- | --- | --- | --- | --- |
| ***rmpA*** | 1.00 | 0.09 | 0.18 | 0.14 | 0.22 | 0.21 |  | 0.22 | -0.03 | -0.04 | 0.11 | 0.03 |
| ***rmpA2*** | 0.09 | 1.00 | 0.55 | 0.04 | 0.44 | 0.66 |  | 0.12 | 0.01 | -0.08 | 0.22 | 0.21 |
| ***iucA*** | 0.18 | 0.55 | 1.00 | 0.03 | 0.20 | 0.74 |  | 0.10 | 0.00 | -0.09 | 0.19 | 0.23 |
| ***iroB*** | 0.14 | 0.04 | 0.03 | 1.00 | 0.26 | 0.00 |  | 0.70 | 0.07 | -0.01 | 0.04 | 0.03 |
| ***peg-344*** | 0.22 | 0.44 | 0.20 | 0.26 | 1.00 | 0.31 |  | 0.18 | 0.05 | -0.05 | 0.14 | 0.13 |
| ***iutA*** | 0.21 | 0.66 | 0.74 | 0.00 | 0.31 | 1.00 |  | 0.08 | -0.05 | -0.11 | 0.21 | 0.29 |
| ***magA*** |  |  |  |  |  |  |  |  |  |  |  |  |
| ***aerobactin*** | 0.22 | 0.12 | 0.10 | 0.70 | 0.18 | 0.08 |  | 1.00 | 0.05 | -0.01 | 0.03 | 0.02 |
| ***ybts*** | -0.03 | 0.01 | 0.00 | 0.07 | 0.05 | -0.05 |  | 0.05 | 1.00 | -0.07 | -0.01 | 0.00 |
| ***alls*** | -0.04 | -0.08 | -0.09 | -0.01 | -0.05 | -0.11 |  | -0.01 | -0.07 | 1.00 | -0.17 | -0.18 |
| ***rmkD*** | 0.11 | 0.22 | 0.19 | 0.04 | 0.14 | 0.21 |  | 0.03 | -0.01 | -0.17 | 1.00 | 0.78 |
| ***fim H*** | 0.03 | 0.21 | 0.23 | 0.03 | 0.13 | 0.29 |  | 0.02 | 0.00 | -0.18 | 0.78 | 1.00 |
| ***kfu*** | -0.17 | 0.00 | 0.03 | 0.10 | -0.12 | -0.05 |  | -0.04 | 0.03 | 0.10 | 0.02 | 0.14 |
| ***entB*** | 0.13 | 0.20 | 0.23 | 0.04 | 0.15 | 0.30 |  | 0.03 | 0.05 | -0.15 | 0.58 | 0.69 |
| ***wab G*** | 0.10 | 0.19 | 0.21 | 0.03 | 0.12 | 0.26 |  | 0.02 | 0.03 | -0.20 | 0.77 | 0.91 |
| **K1** |  |  |  |  |  |  |  |  |  |  |  |  |
| **K2** | -0.03 | -0.06 | -0.06 | -0.01 | -0.04 | -0.08 |  | -0.01 | -0.14 | -0.01 | 0.03 | 0.02 |
| **K5** | -0.09 | -0.10 | -0.12 | -0.03 | -0.11 | -0.17 |  | -0.02 | 0.00 | -0.03 | -0.56 | -0.70 |
| **K20** | -0.03 | 0.12 | 0.10 | -0.01 | -0.04 | 0.08 |  | -0.01 | 0.05 | -0.01 | 0.03 | 0.02 |
| **K54** |  |  |  |  |  |  |  |  |  |  |  |  |
| **K57** | 0.14 | 0.04 | 0.03 | 0.49 | 0.10 | 0.00 |  | 0.70 | 0.07 | -0.01 | 0.04 | 0.03 |
| **K non-typeable** | 0.03 | 0.05 | 0.08 | -0.18 | 0.07 | 0.14 |  | -0.28 | 0.00 | 0.03 | 0.43 | 0.55 |

Notes: Blank space indicates that no modified genotype is detected

**Continued form** **Date7** Pearson correlation coefficients of genes in CRKP

| **Gene** | ***kfu*** | ***entB*** | ***wab G*** | **K1** | **K2** | **K5** | **K20** | **K54** | **K57** | **K non-typeable** |
| --- | --- | --- | --- | --- | --- | --- | --- | --- | --- | --- |
| ***rmpA*** | -0.17 | 0.13 | 0.10 |  | -0.03 | -0.09 | -0.03 |  | 0.14 | 0.03 |
| ***rmpA2*** | 0.00 | 0.20 | 0.19 |  | -0.06 | -0.10 | 0.12 |  | 0.04 | 0.05 |
| ***iucA*** | 0.03 | 0.23 | 0.21 |  | -0.06 | -0.12 | 0.10 |  | 0.03 | 0.08 |
| ***iroB*** | 0.10 | 0.04 | 0.03 |  | -0.01 | -0.03 | -0.01 |  | 0.49 | -0.18 |
| ***peg-344*** | -0.12 | 0.15 | 0.12 |  | -0.04 | -0.11 | -0.04 |  | 0.10 | 0.07 |
| ***iutA*** | -0.05 | 0.30 | 0.26 |  | -0.08 | -0.17 | 0.08 |  | 0.00 | 0.14 |
| ***magA*** |  |  |  |  |  |  |  |  |  |  |
| ***aerobactin*** | -0.04 | 0.03 | 0.02 |  | -0.01 | -0.02 | -0.01 |  | 0.70 | -0.28 |
| ***ybtS*** | 0.03 | 0.05 | 0.03 |  | -0.14 | 0.00 | 0.05 |  | 0.07 | 0.00 |
| ***alls*** | 0.10 | -0.15 | -0.20 |  | -0.01 | -0.03 | -0.01 |  | -0.01 | 0.03 |
| ***rmkD*** | 0.02 | 0.58 | 0.77 |  | 0.03 | -0.56 | 0.03 |  | 0.04 | 0.43 |
| ***fim H*** | 0.14 | 0.69 | 0.91 |  | 0.02 | -0.70 | 0.02 |  | 0.03 | 0.55 |
| ***kfu*** | 1.00 | 0.11 | 0.13 |  | -0.04 | -0.11 | -0.04 |  | -0.05 | 0.14 |
| ***entB*** | 0.11 | 1.00 | 0.77 |  | 0.03 | -0.69 | 0.03 |  | 0.04 | 0.53 |
| ***wab G*** | 0.13 | 0.77 | 1.00 |  | 0.02 | -0.77 | 0.02 |  | 0.03 | 0.61 |
| **K1** |  |  |  |  |  |  |  |  |  |  |
| **K2** | -0.04 | 0.03 | 0.02 |  | 1.00 | -0.02 | -0.01 |  | -0.01 | -0.28 |
| **K5** | -0.11 | -0.69 | -0.77 |  | -0.02 | 1.00 | -0.02 |  | -0.03 | -0.80 |
| **K20** | -0.04 | 0.03 | 0.02 |  | -0.01 | -0.02 | 1.00 |  | -0.01 | -0.28 |
| **K54** |  |  |  |  |  |  |  |  |  |  |
| **K57** | -0.05 | 0.04 | 0.03 |  | -0.01 | -0.03 | -0.01 |  | 1.00 | -0.39 |
| **K non-typeable** | 0.14 | 0.53 | 0.61 |  | -0.28 | -0.80 | -0.28 |  | -0.39 | 1.00 |

Notes: Blank space indicates that no modified genotype is detected

**Date 8** The common ST types of CSKP and CRKP

| **Group** | **Common ST classification [n (%)]** |
| --- | --- |
| CSKP  （n=213） | ST23 [27（12.7）]、ST65 [11（5.2）]、ST286 [10（4.7）]、ST1764 [10（4.7）]、  ST412 [9（4.2）]、ST1429 [7（3.3）]、ST290 [7（3.3）]、ST109 [6（2.8）]、  ST1308 [6（2.8）]、ST1770 [6（2.8）]、ST2793 [6（2.8）]… |
| CRKP  （n=150） | ST11 [75（50.0）]、ST437 [21（14.0）]、ST15 [16（10.7）]、ST290 [11（7.3）]、  ST307 [5（3.3）]、ST4 [3（2.0）]、ST37 [3（2.0）]、ST35 [2（1.3）]、  ST412 [2（1.3）]、ST3113 [2（1.3）]… |

**Date 9** Phylogenetic tree data

| **Source** | **Target** | **Count** |
| --- | --- | --- |
| CSKP | hmKP | 90 |
| CSKP | n-hmKP | 123 |
| CRKP | n-hmKP | 141 |
| CRKP | hmKP | 9 |
| hmKP | hvKP | 62 |
| n-hmKP | n-hvKP | 261 |
| n-hmKP | hvKP | 3 |
| hmKP | n-hvKP | 37 |
| hvKP | K1 | 31 |
| hvKP | K2 | 11 |
| hvKP | K5 | 4 |
| hvKP | K20 | 4 |
| hvKP | K54 | 3 |
| hvKP | K57 | 9 |
| hvKP | K-non | 3 |
| n-hvKP | K1 | 4 |
| n-hvKP | K2 | 10 |
| n-hvKP | K5 | 23 |
| n-hvKP | K20 | 4 |
| n-hvKP | K54 | 5 |
| n-hvKP | K57 | 3 |
| n-hvKP | K-non | 249 |
| K1 | ST23 | 27 |
| K1 | ST367 | 1 |
| K1 | ST412 | 1 |
| K1 | ST700 | 1 |
| K1 | ST793 | 1 |
| K1 | ST882 | 1 |
| K1 | ST1265 | 1 |
| K1 | ST2159 | 1 |
| K-non | ST11 | 74 |
| K5 | ST11 | 1 |
| K5 | ST437 | 7 |
| K-non | ST437 | 14 |
| K-non | ST15 | 18 |
| K-non | ST290 | 18 |
| K-non | ST65 | 5 |
| K2 | ST65 | 5 |
| K54 | ST65 | 1 |
| K57 | ST412 | 10 |
| K-non | ST268 | 4 |
| K2 | ST268 | 1 |
| K20 | ST268 | 5 |
| K-non | ST1764 | 10 |
| K-non | ST1429 | 7 |
| K5 | ST109 | 2 |
| K-non | ST109 | 4 |
| K5 | ST1308 | 1 |
| K-non | ST1308 | 5 |
| K-non | ST1770 | 6 |
| K-non | ST2793 | 6 |
| K2 | ST25 | 3 |
| K5 | ST25 | 2 |
| K2 | ST29 | 1 |
| K54 | ST29 | 4 |
| K-non | ST36 | 5 |
| K-non | ST307 | 5 |
| K2 | ST1039 | 1 |
| K54 | ST1039 | 2 |
| K-non | ST1039 | 2 |
| K2 | ST1266 | 1 |
| K-non | ST1266 | 4 |
| K-non | ST45 | 3 |
| K5 | ST45 | 1 |
| K-non | ST176 | 4 |
| K2 | ST1049 | 1 |
| K5 | ST1049 | 3 |
| K-non | ST3106 | 4 |
| K2 | ST375 | 2 |
| K2 | ST5559 | 2 |
| K2 | others | 4 |
| K5 | ST485 | 2 |
| K5 | ST515 | 2 |
| K5 | ST519 | 2 |
| K5 | others | 4 |
| K54 | ST3405 | 1 |
| K57 | ST5107 | 1 |
| K57 | ST1 | 1 |
| K-non | others | 54 |
